# Nano-Biosensor for Monitoring the Neural Differentiation of Stem Cells

**DOI:** 10.3390/nano6120224

**Published:** 2016-11-28

**Authors:** Jin-Ho Lee, Taek Lee, Jeong-Woo Choi

**Affiliations:** 1 Department of Chemical and Biomolecular Engineering, Sogang University, 35 Baekbeom-ro (Sinsu-dong), Mapo-gu, Seoul 121-742, Korea; jino@sogang.ac.kr (J.-H.L.); nanotlee@gmail.com (T.L.); 2 Institute of Integrated Biotechnology, Sogang University, 35 Baekbeom-ro (Sinsu-dong), Mapo-gu, Seoul 121-742, Korea

**Keywords:** biosensor, neural differentiation, stem cell, nanostructure, optical detection method, electrochemical detection method

## Abstract

In tissue engineering and regenerative medicine, monitoring the status of stem cell differentiation is crucial to verify therapeutic efficacy and optimize treatment procedures. However, traditional methods, such as cell staining and sorting, are labor-intensive and may damage the cells. Therefore, the development of noninvasive methods to monitor the differentiation status in situ is highly desirable and can be of great benefit to stem cell-based therapies. Toward this end, nanotechnology has been applied to develop highly-sensitive biosensors to noninvasively monitor the neural differentiation of stem cells. Herein, this article reviews the development of noninvasive nano-biosensor systems to monitor the neural differentiation of stem cells, mainly focusing on optical (plasmonic) and eletrochemical methods. The findings in this review suggest that novel nano-biosensors capable of monitoring stem cell differentiation are a promising type of technology that can accelerate the development of stem cell therapies, including regenerative medicine.

## 1. Introduction

The intrinsic ability of stem cells to undergo continual proliferation and differentiation into any given cell type offers a promising therapeutic strategy for regenerative medicine [1,2,3,4,5,6]. The self-renewal capability of stem cells is critical to generate a sufficient quantity of cells for large-scale cell-based applications, as well as differentiation into defined lineages with mature function to treat tissue-specific degenerative disease [7,8,9]. One major obstacle to the clinical translation of stem cell-based therapy involves the identification of a terminal differentiation state of the stem cell or its tumorigenic potential [10]. Specifically, accurately monitoring cell differentiation is extremely important in treating devastating neural diseases, including Parkinson’s disease (PD), Alzheimer’s disease and spinal cord injuries [11]. For example, PD is primarily caused by a decrease in dopaminergic neurons in the substantia nigra at the mid brain, so stem cell-derived dopaminergic neurons are typically required for transplantation [12,13,14,15].

It is critical to identify and characterize specifically-differentiated cells prior to clinical translation, and conventional methods such as fluorescence-based methods and biomolecular analyses are used to define the differentiation status as well as to distinguish undifferentiated stem cells from differentiated neuronal and glial cells [16,17,18]. Although these techniques are highly sensitive and can be used to precisely determine the status of differentiated cells, such methods also tend to be time-consuming, laborious, potentially toxic and, most importantly, require destructive steps including cell fixation or lysis, making them unsuitable for clinical use. Thus, there is a pressing need to obtain highly-sensitive, noninvasive approaches to effectively identify stem cell fate (into undifferentiated and differentiated states) in order to fulfill the potential of stem cell-based therapies.

Recent advances in nanotechnology have led to the development of biosensors with improved sensitivity and performance. The unique properties and appropriate surface modifications of various nanomaterials that have been utilized in the development of nano-biosensors allow for the diagnoses with molecular markers with extremely high sensitivities [19,20]. For instance, the distinct function of metal nanomaterials (e.g., Au, Ag, etc.), including enhanced surface plasmon resonance, have directed the development of several novel optical biosensors. Furthermore, high surface-to-volume ratios of nanomaterial-facilitated enhanced performance in sensing systems can be achieved by providing more active regions as well [21]. Accordingly, nano-biosensors have attracted attention for applications where extremely low concentrations of small molecules need to be analyzed. For example, the major issue in analyzing living cells is their complex structure and environment, which makes it difficult to reliably identify differences at the molecular level. Thus, novel approaches to develop extremely sensitive and accurate biosensors to monitor the molecular changes in the presence of a complex cellular background will be of great benefit for live cell analysis, including monitoring the differentiation status of the stem cells [22,23,24].

A major goal of this review is to outline the recent progress in non-invasive monitoring methods for neural stem differentiation, provide brief and concise information for engineers, and promote interest in live cell study applications. We first provide an overview of the effects of functional nanomaterials on biosensors and then highlight newly-developed nano-biosenors to monitor neural differentiation, mainly focusing on optical (plasmonic) and electrochemical methods.

## 2. Role of Nanotechnology in Developing Biosensors

Biosensors are powerful tools that analyze biomolecular interactions in bio/chemical and environmental analyses [25]. The structure of a typical biosensor consists of a transducer comprised of a biological recognition component as a key feature. The interaction between the target molecule and the biorecognition component is converted into a measurable signal by the transducer. Researchers from various disciplines, including physics, chemistry, biology and engineering, have expended tremendous efforts to improve the performance of biosensors. In the quest to improve the performance of existing and potential biosensors, integrating nanomaterials is a promising approach due to their unique chemical and physical properties (e.g., electrical and optical properties, among others). For example, an electrochemical sensor system has an electrode that is critical to the sensor performance since the reactions mostly occur in close proximity to the electrode surface. Based on the functional properties of the materials that are utilized, their surface morphology and modification greatly influences the sensing ability. Therefore, platinum, gold, and carbon-based nanomaterials have attracted a significant amount of attention due to their higher conductivity, biocompatibility, and larger surface area [26]. In further detail, the higher surface-to-volume ratio of the nanomaterial enhances the electrical properties of the electrode by increasing the active surface that is exposed to external fluids. In addition, since the dimensions of the nanostructures are similar to the size of the target molecules, the capture efficiency can be improved, which in turn leads to an increase in sensitivity [27,28].

**Figure 1:**
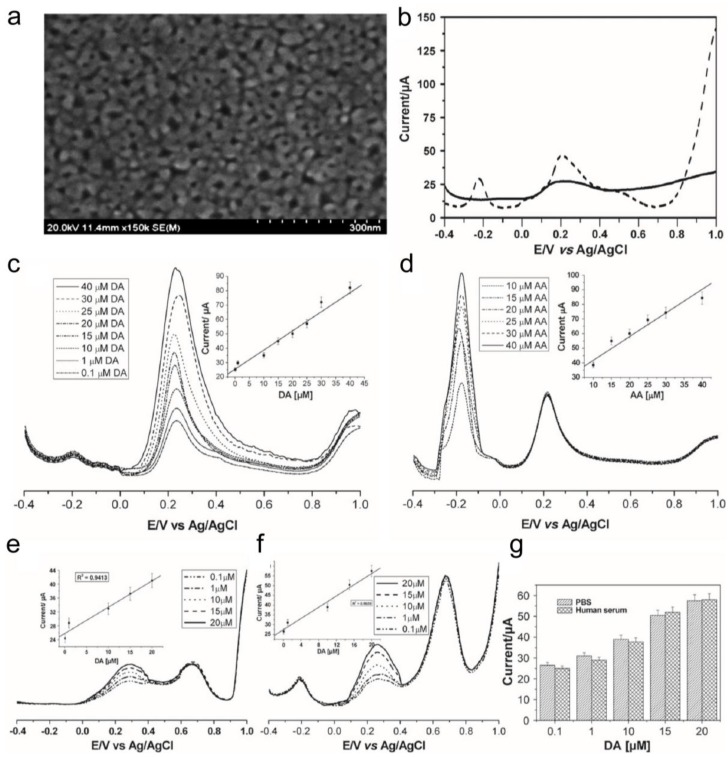
(**a**) Scanning Electron Microscopy (SEM) image of a nanoporous gold film (NPGF)-based electrode surface. Differential pulse voltammetry (DPV) results for (**b**) a mixture of ascorbic acid (AA) and dopamine (DA) at (solid line) bare Au and (dashed line) NPGF electrode; (**c**) Varying concentrations of DA (0.1–40 μM) in the presence of AA (5 μM). Inset: Linear plot of the anodic current peak as a function of the DA concentration (**d**) varying concentrations of AA (10–40 μM) in the presence of DA (5 μM). Inset: Linear plot of anodic current peak as a function of AA concentration; (**e**) Varying concentrations of DA in the presence of uric acid (UA) (500 μM). Inset: Linear plot of anodic current peak as a function of DA concentration; (**f**) Varying concentrations of DA in the presence of AA (5 μM) and UA (1 mM). Inset: Linear plot of anodic current peak as a function of DA concentration; (**g**) Anodic current peak corresponding to oxidation of varying concentrations of DA (0.1–20 μM) in the presence of AA (5 μM) and UA (0.5 mM) in both human serum and phosphate buffered saline (PBS) buffer. (Modified from Ref. [29] with permission, Copyright 2010 Elsevier (Amsterdam, The Netherlands)).

One further issue regarding biosensors is that the signal is interfered by biological/chemical substances that exist in a complex biological matrix. Relatedly, El-Said et al. proposed a 3-D nanoporous gold film (NPGF)-modified electrode to distinguish a dopamine signal from the presented interfering materials (Figure 1a). It should be noted that dopamine is one of the essential markers to analyze Parkinson’s disease and that it can also be used as a biomarker to monitor stem cell differentiation into dopaminergic neuron [29,30].

As shown in Figure 1b, a broad and overlapped peak signal was obtained for the mixture of DA and AA on the bare gold electrode, in which it was difficult to distinguish the independent oxidation potential peaks of AA and DA. Comparably, according to the presence of a nanoporous structure that can provide a higher surface-to-volume ratio and faster electron transfer on a simultaneously-trapped electroactive species inside of the nanopore [31], two well-defined oxidation peaks for DA and AA could be observed at the NPGF electrode. In addition, DA and AA were simultaneously determined in a mixture of DA and AA at the NPGF electrode. Figure 1c,d show an increase in the DPV signal in accordance with an increase in the concentration of DA (0.1–40 μM) in the presence of AA (5 μM) as well as an increase in the concentration of AA (10–40 μM) in the presence of DA (5 μM). In parallel, NPGF also exhibited reliability even in human serum (1%) solution with highly concentrated interfering materials, such as AA (10 μM) and UA (1 mM). These results indicate that NPGF is more suitable compared to a bare gold electrode in detecting an extremely low concentration of DA in a complex biological matrix. Taking advantage of the unique physicochemical properties of the nanomaterial, which is not limited to the electrochemical properties discussed above, several efforts have been undertaken to develop biosensors with improved properties.

## 3. Electrochemical and Electrical Detection System to Monitor Stem Cell Differentiation

**Figure 2:**
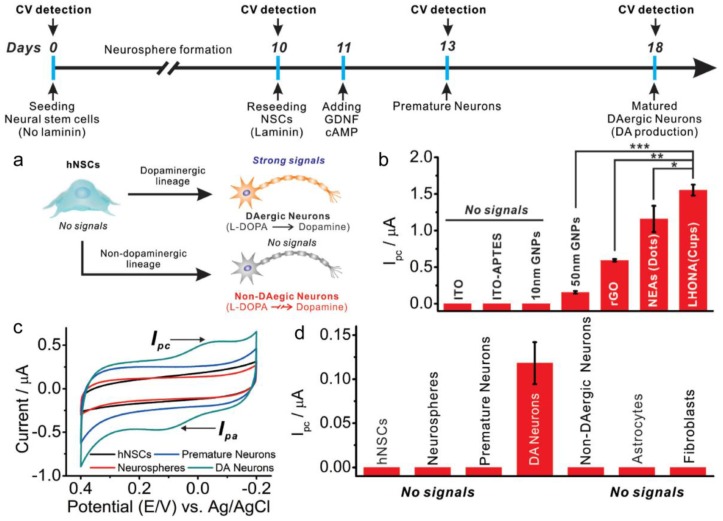
(**a**) Schematic diagram depicting the conversion of human neural stem cells (hNSCs) into dopaminergic (DAergic) and non-DAergic neurons; (**b**) The oxidation peak intensities of dopamine obtained from the CV with various electrodes (Student’s *t*-test, *N* = 3, * *p* < 0.05, ** *p* < 0.01, *** *p* < 0.001) (**c**) Cyclic voltammogram obtained from cells undergoing differentiation into DAergic Neurons. The result only show completely matured DAergic neurons that reveal clear redox peaks compared to hNSCs, neurospheres, and premature neurons; (**d**) Oxidation peak intensities obtained from (**c**) and other types of cells (astrocytes and fibroblasts) (Modified from Ref. [33] with permission, Copyright 2015 WIELY-VCH Verlag GmbH, Berlin, Germany).

Accordingly, the ability to detect a neurotransmitter, such as dopamine, can be useful as a biomarker to distinguish the differentiation status of stem cell differentiation [32,33]. If a very low concentration of dopamine can be detected using an electrochemical method, then it could be possible to fabricate an effective in situ monitoring biosensor for dopaminergic stem cell differentiation. Recently, Kim et al. proposed using a large-scale homogeneous gold nanocup array as a platform to monitor dopaminergic differentiation of human neural stem cells (hNSC) using an electrochemical technique [33]. (Figure 2) In this study, a large-scale homogeneous gold nanocup-electrode array (LHONA) scale is fabricated on an indium tin oxide (ITO) substrate using laser interference lithography (LIL) and electrochemical deposition (ECD). In the first step, a well-defined photoresist (PR) grid nanopattern is prepared onto the ITO surface via LIL as the template. Then, the gold colloid (HAuCl_4_) is electrochemically deposited and adequate selection of the gold concentration, surfactant concentration and electrochemical deposition time are required to fabricate the homogeneous gold nanocup array. Furthermore, the results show that the intensities of the reduction peaks of dopamine (10 µM), which are monitored via cyclic voltammetry (CV), correspond to various substrates (bare ITO, 10 nm Au nanoparticle (NP) on ITO, 50 nm Au NP and reduced graphene oxide (rGO)), and a linear correlation exists between the dopamine concentration and reduction peak signal intensity. The results indicate that the fabricated LHONA shows the highest reduction signal linearity (0.3−3 × 10 µM). Based on the prepared LHONA electrode, the PC12 cells are cultured on a LHONA electrode to observe the improvement in cell adhesion and proliferation, as compared to a bare Au substrate and tissue culture plate (TCP). The results show that the total surface area of the PC12 cells is larger than 88.9% of the bare gold surface and 12.4% of the TCP surface, respectively. Also, despite washing, the PC12 cells that adhere to the LHONA electrode do not wash out when compared to TCP. This result is likely due to the improvement in cell adhesion, proliferation and high biocompatibility provided by the nanostructure.

Subsequently, CV is carried out for PC12 cells and hNSC cells immobilized in the LHONA electrode to detect the dopamine released from the individual cells, respectively. In particular, the detection of dopamine redox signals from the hNSC-derived dopaminergic neurons is very important for clinical tests, such as Parkinson’s disease or attention deficit hyperactivity disorder (ADHD) [34,35]. For this reason, the ReNcell-VM human neural progenitor cell line is used to generate dopaminergic neurons with a high level of differentiation. Only dopaminergic neurons can be detected by the dopamine redox signals using CV since nondopaminergic neurons and undifferentiated hNSCs (neurospheres and premature neurons) do not show redox signals using CV (Figure 2a). Also, to assess the dopaminergic neuron more clearly, the cells are stained in a tyrosine hydroxylase test (TH). Figure 2b displays various oxidation peaks obtained from the DA detection of LHONA and other types of electrodes, such as bare ITO, Au NP on ITO, nanoelectrode array (NEAs, dot-like structure) and reduced graphene oxide (rGO). The result shows that the oxidation peak of LHONA is excellent when compared to others. Using LHONA, only the dopaminergic neuron from the hNSC reveals clear redox peaks (Figure 2c,d). Thus, the gold nanostructure-based biosensor can provide a powerful detection platform for stem cell differentiation.

In parallel, an electrical measurement technique has also been used to observe the neural differentiation of stem cells. In particular, the electric cell-substrate impedance sensing (ECIS) technique is a very interesting tool to non-invasively monitor stem cell differentiation. When the alternating current (AC) impedance is applied to the cell-coated electrode, the cell membranes act as dielectric materials according to the cell shape and morphology [36,37]. This change produces the frequencies of the cell-coated electrode that are recorded as a function of time in real time. This technique was also applied to monitor the adipogenic and osteogenic differentiation of bone marrow-derived stem cells, confirming the morphological changes using ECIS [38,39].

Lin et al. reported on an electric cell-substrate impedance sensing (ECIS) system with field effect transistor (SiNW FET) to detect the differentiation of PC12 cells on silicon nanowire electrodes in real-time [40]. They monitored cell morphology and growth during neuronal differentiation for 5 to 7 days using a SiNW-coated FET device through E4980A precision LCR meter (Agilent Technologies, Santa Clara, CA, USA). The PC12-SiNW FET system showed changes in the impedance values during cell growth and differentiation due to a negatively charged cell surface and SiNW resistance at the cell/SiNW interface. When the PC12 cells were differentiated, the impedance magnitude increased to become more negative. This result revealed that the ECIS technique with the nanostructure can provide a platform for stem cell differentiation in real time. Moreover, the impedance technique can be applied to detect the cellular response, such as cell proliferation, viability and toxicity, to the nanostructure [41,42]. So far, the ECIS technique with the given nanostructure for stem cell differentiation has not been reported. However, if the stem cell on the nanostructure-modified electrode was introduced into the ECIS system, then the differentiation efficiency of the stem cell is expected to increase as a result of the nanostructure. This will provide a useful non-invasive tool as a biosensor to monitor stem cells.

## 4. Optical Detection System to Monitor Stem Cell Differentiation

Rapid advances in the field of optical biosensors with nanostructures have led to the development of ultra-sensitive optical biosensors to detect antigens [43], viruses [44] and neurotransmitters [45]. In particular, surface-enhanced Raman spectroscopy (SERS) has several advantages in the detection of a cell differentiation signal, including that it is a non-invasive, label-free technique with high sensitivity and well-defined nanostructures [46,47]. To the best of our knowledge, the mechanism for the SERS effect is derived from the electromagnetic field and chemical enhancement [21,48]. The fabrication of a well-defined nanostructure is essential to measure the SERS signal from the few target molecules. Since the improvement in the SERS signal is affected by the chemical composition, surface roughness and size of the substrate, this enables the detection of a few molecules with fluctuating spectra [49,50]. This non-destructive and highly-sensitive technique can provide a platform to detect virus pathogens or cell differentiation of stem cells at a single level for biomedical application. For example, Shanmukh et al. suggested a pathogen detection biosensor composed of an Ag nanorod array using a SERS technique [51]. The Ag nanorod array electrode is prepared using oblique angle vapor deposition (OAVD) to provide a SERS hot spot. Then, different viruses (adenovirus, rhinovirus and HIV) show different SERS spectra corresponding to the composition of virus such as DNA, RNA and proteins. Thus, the SERS with a highly-ordered nanostructure can be used as a detection platform to distinguish small, specific molecules.

**Figure 3:**
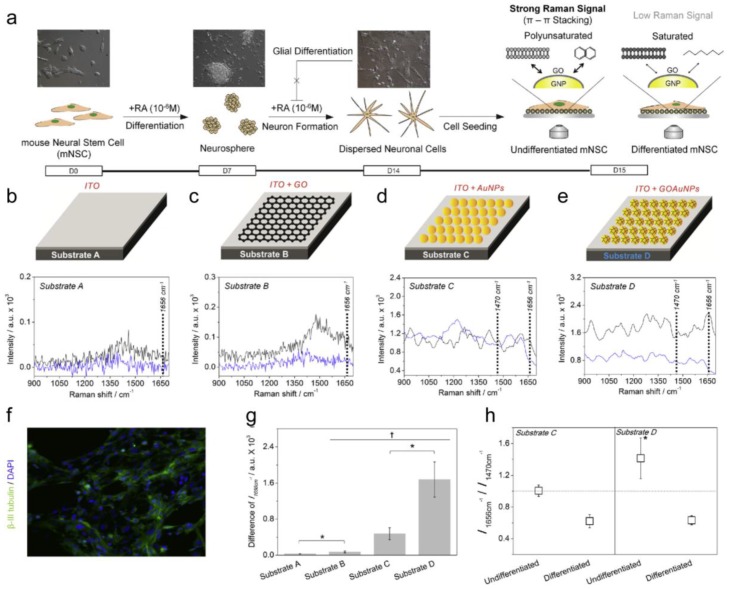
(**a**) Schematic diagram representing the method to detect the undifferentiated and differentiated state of the mouse neural stem cells (NSCs) using 3D GO-encapsulated gold nanoparticles. Raman spectra of (Full-size image (2 K)) undifferentiated or (Full-size image (2 K) differentiated mNSCs on (**b**) Substrate A: indium tin oxide (ITO); (**c**) Substrate B: GO coated ITO; (**d**) Substrate C: AuNP coated ITO and (**e**) Substrate D: GO-encapsulated AuNP coated ITO; (**f**) Confocal fluorescence images of differentiated mNSCs on Substrate D showing the successful differentiation of mNSCs to neuronal cells; (**g**) Intensity difference of Raman peaks at 1656 cm^−1^ (C double bond; length as m-dashC bond) achieved from undifferentiated mNSCs subtracted by differentiated cells († *p* < 0.05, *N* = 3, ANOVA test and * *p* < 0.05, Student’s *t*-test); (**h**) Relative values of the Raman intensity at 1656 cm^−1^ divided by the intensity at 1470 cm^−1^. All the Raman spectra of the mNSCs were subtracted by the Raman spectra of the same substrates without cells to eliminate the background signals. The results are the medians of the Raman signals obtained from ten different spots. (Modified from Ref. [24] with permission, Copyright 2013 Elsevier).

Kim et al. measured the differentiation potential of neural stem cells (NSCs) based on SERS [24]. In detail, a new material consisting of 3D-structured graphene oxide (GO)-encapsulated gold nanoparticles was developed to deliver a double improvement in the metal nanoparticles and graphene oxide on the SERS signals. The difference in the Raman signal obtained from the undifferentiated NSCs on the graphene oxide (GO)-encapsulated gold nanoparticles and normal metal structures was of a factor of about 3.5, and these were clearly distinguishable from differentiated cells. It should be noted that without the help of the gold nanoparticle, no observable signal differences were obtained from undifferentiated and differentiated NSCs. The Raman intensity at 1656 cm^−1^ correlated to the number of C=C bond was found to match with the differentiation state of the NSCs (Figure 3).

**Figure 4:**
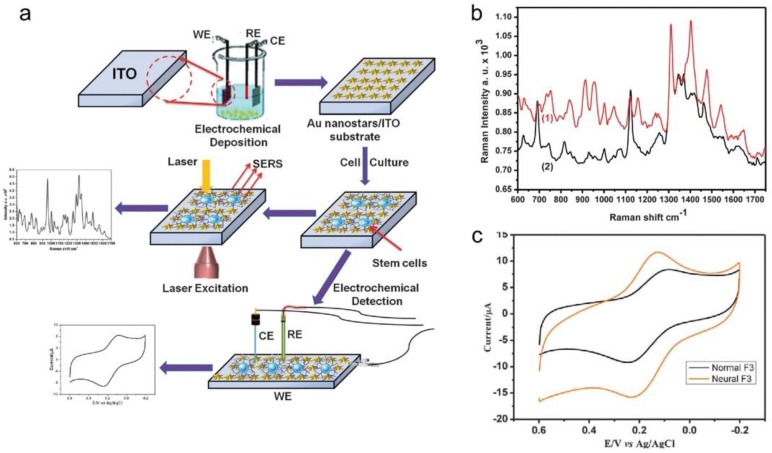
(**a**) Schematic diagram showing the spectro-electrochemical-based neural stem cell differentiation monitoring sensor on a gold nanostar array; (**b**) Raman spectrum for (1) undifferentiated and (2) differentiated HB1.F3 cells within the range of 600 cm^−1^ to 1750 cm^−1^; (**c**) cyclic voltamogram for differentiated and undifferentiated HB1.F3 cells. (Modified from Ref. [52] with permission, Copyright 2015 The Royal Society of Chemistry, London, UK).

El-said et al. proposed a cell-based biosensor to monitor the differentiation of neural stem cells on gold nanostar arrays using a combinatory SERS technique and an electrochemical technique (Figure 4a) [52]. In this study, the gold nanostar array is fabricated onto an ITO substrate using electrochemical deposition (ECD) with a reduction in an HAuCl_4_ aqueous solution and PEG. The fabricated gold nanostar array on the ITO substrate is used in cell culturing, SERS and electrochemical experiments. The result shows the SEM results of a fabricated Au nanostar with a different morphology. These results indicate that the different shape and size of the gold nanostar corresponds to the concentration of the HAuCl_4_. The results show the ultraviolet–visible (UV-Vis) spectra of fabricated Au nanostars on the ITO substrate. Two plasmon absorption peaks around 553 nm and 704 nm are revealed in the spectra. The first peak at 553 nm originates from the transverse electronic oscillation and another peak (704 nm) originates from the longitudinal oscillation of the electrons. These plasmon peaks can serve as SERS hot spots to monitor the stem cell differentiation [52,53]. Figure 4a depicts the Au nanostar array on the ITO substrate to monitor the differentiation of the stem cells using SERS and CV. Figure 4b shows the Raman spectra of undifferentiated HB1.F3 cells (1) and differentiated HB1.F3 cells (2) on the Au nanostar structure. Furthermore, CV experiments are conducted to observe the differentiation of the HB1.F3 cell (Figure 4c). Also, the differentiation of mouse neural stem cells is investigated by SERS for 28 days. The SERS technique can also be used to monitor the differentiation level from the protein expression in real time according to the incubation time on the well-oriented gold nanostar substrate. Such results demonstrate that the optical properties of a well-ordered gold nanostar array can be used to detect the differentiation of neural stem cells without an additional marker [52].

On the other hand, different optical techniques, such as those using localized surface plasmon resonance-based detection systems, were introduced to monitor the changes in the cell signal and status in the cancer cell on the nanostructure. The exosomes are nanovesicle composed of a phospholipid that can be used to determine the cancer cell status. Presumably, monitoring the exosome is a promising new approach to identify neural stem cell differentiation [54]. So far, nano-based biosensors for exosome analysis have been used in the context of cancer cell studies. Im et al. reported on the monitoring method of exosomes profiling in ovarian cancer cells on periodic nanohole arrays using transmission surface plasmon resonance. The specific exosome was bound to the nano-plasmonic exosome assay chip using an antibody, and then this binding event changed its local refractive index to be recorded by measuring the wavelength shift (△*λ*) or the change in intensity (△*p*). The fabricated nano-plasmonic exosome assay chips monitor the expression of an ovarian cancer protein marker CD24 and EpCAM with a high-throughput and were rapidly compared to western blot analysis or ELISA assays. As such, the combination of optical techniques and nanostructures can bring new insights to monitor cell signal changes [55].

## 5. Outlook

Stem cells are a promising resource for tissue engineering and regenerative medicine application. Their unique ability to replicate and differentiate into specific lineages make them suitable for use in certain tissue engineering applications. The major challenges to stem cell therapy are to achieve specified stem cell differentiation with a high yield toward clinically relevant lineages with mature functions and to assess their tumorigenic potential. However, traditional methods to determine cell differentiation (e.g., cell staining and sorting) are labor-intensive, time consuming and mostly cause damage to the cells. Consequently, demand for simple, rapid biosensors with noninvasive techniques are increasing. Although noninvasive methods to identify the differentiation state of the stem cells is a relatively new concept and is in its initial stages, the cell-based biosensors discussed herein also have some positive and negative aspects (Table 1).

In the case of a SERS-based detection system, there are several advantages including rapid signal acquisition, single cell analysis and very high-selectivity compared to fluorescence-based techniques. However, such techniques still have some limitations. For example, there is a need for well-defined nanostructures with hot-spots, expensive optical apparatus and the reliable systems to monitor cell differentiation. In the case of electrochemical-based measurement system, the system itself is very simple, inexpensive and does not need additional labeling.

However, the electrochemical characteristics of the cells are mainly based on cell viability or change in the secretases level, which is not direct for stem-cell differentiation. Also, the redox signal from the cell differentiation is as yet undefined. This is a challenge to conduct single cell level analysis with an electrochemical-based measurement system as well. While currently proposed cell-based nano-biosensors exhibit effective properties, including noninvasive in situ monitoring tool for stem-cell differentiation, interdisciplinary research efforts are still required to achieve more effective stem-cell-based therapies that target incurable diseases/disorders. In the future, the design and fabrication of nanostructures will need to be integrated with multifunctional biochemical modifications to simultaneously control multiple aspects of an in vitro environment and fine tune specific terminal lineage commitments while maintaining the unique properties of detection systems.

## Figures and Tables

**Table 1:** Comparison of four techniques based on their advantages and disadvantages.

Detection Platform	Advantage	Disadvantage
Optical (SERS)	Narrow band spectra Rapid signal acquisition time High sensitivity	Requires reliable system (Highly ordered substrate)
Optical (LSPR)	High sensitivity Multiple sample analysis	Difficult to distinguish different binding events in sample mixtures
Electrochemical (CV/DPV)	Simple to operate Label-free analysis	Not complementary defined where redox signal originates
Electrical (ECIS)	Real-time signal acquisition Label-free analysis	Time consuming Undesired signal from environment
